# Special Issue “The Ability–Personality Integration”

**DOI:** 10.3390/jintelligence7020013

**Published:** 2019-06-25

**Authors:** Roberto Colom, Doreen Bensch, Kai T. Horstmann, Caroline Wehner, Matthias Ziegler

**Affiliations:** 1 Departamento de Psicología Biológica y Salud, Facultad de Psicología, Universidad Autónoma de Madrid, 28049 Madrid, Spain; 2 Institut für Psychologie, Humboldt-Universität zu Berlin, Rudower Chaussee 18, 12489 Berlin, Germany; benschdx@hu-berlin.de (D.B.); horstmak@hu-berlin.de (K.T.H.); wehnerca@hu-berlin.de (C.W.)

Humans display varied behaviors, and scientists put enormous research efforts into finding explanations for them. For instance, some humans seem paralyzed when they must talk in front of a crowded room, whereas other humans seem eager to do it. The explanations to phenomena such as this may involve unobservable traits presumably underlying such observed behavioral differences. In the above example, one explanation is that the people in the first group are shy, but they could also feel terrified, because they know their verbal ability is below the level required to deliver the expected sophisticated and convincing message to the audience. The second group of humans could comprise sensation seekers who pay considerably less attention to their limited cognitive abilities.

Finding sound answers requires sustained research, but the scientific method is more analytic than synthetic. As underscored by E. O. Wilson in *Consilience* [1], “complexity is what interests scientists in the end, not simplicity. Reductionism is the way to understand it. The love for complexity without reductionism is art; the love for complexity with reductionism makes science” (p. 59).

Multivariate approaches may help but may be also dangerous. On the one hand, it is reasonable to expect multiple causes of observed behavioral differences in the same situation, but these causes may change in their relevance for different individuals. On the other hand, the availability of large data sets and infinite ways to analyze them require strong theoretical assumptions that help researchers separate signal from noise.

This Special Issue addresses the key question of the interaction (and maybe the integration) between two crucial families of psychological traits, namely, intelligence and personality. As noted by Sackett et al. in their revision of 100 years of individual differences research for the *Journal of Applied Psychology* [2], there are several issues addressed by individual differences research: (1) human evolutionary, genetic, and situational origins; (2) their dimensionality (how we can summarize behavioral differences); (3) their measurement; (4) their stability; and (5) their practical applications. These researchers focused on three domains: (1) abilities, skills, and knowledge; (2) personality; and (3) interests. However, they did not strive to integrate these different but undoubtedly related domains. In fact, there seems to be a divide between researchers interested in such traits as the ‘Big Five’ and those interested in cognitive abilities. This is even more surprising when considering that both can be characterized by the key elements of a definition of personality recently proposed by experts in the field [3]: “A person’s characteristic pattern of behaviours in the broad sense (including thoughts, feelings, and motivation)” (p. 527). Thus, more fitting labels might be the sometimes-used terms of cognitive and non-cognitive traits. However, such labels have the distinct problem that the latter implies that traits such as the Big Five are non-cognitive in nature, which clearly is not the case.

The current Special Issue was announced with the hope of fostering the integration of the unfortunately disparate research fields. The reader will find 11 articles devoted to the interplay between intelligence and personality constructs. Within this general framework, the authors address varied topics, for instance, how cognitive and personality factors are related from childhood to early adulthood [4], the prospective prediction of intelligence and personality from maltreatment and externalizing behaviors experienced in childhood [5], how intelligence and personality contribute to forecast career potential [6], the prediction of scholastic achievement using intelligence and personality variables [7], and the contribution of cognition and personality to ‘perspective taking’ (the ability to infer other peoples’ mental states) [8].

Beyond the enumerated thematic topics, for this Special Issue, we also explicitly invited the submission of theory papers, for instance, the relevance of the Brunswik symmetry for a proper study of the interplay between intelligence and personality—the correlation values between both constructs are remarkably different across levels of generality [9]; the relations between intelligence and personality at the facet level [10]; how we view intelligence–personality relations [11]; the nature of our measurements of intelligence and personality [12]; the relevance of broadening the way we measure personality to study its relationships with intelligence constructs [13]; and the developmental interplay between openness and ability in a microlevel extension of the Openness-Fluid-Crystallized-Intelligence (OFCI) model [14].

Perhaps one of the main conclusions that might be derived from the whole set of articles is that there are conceptually relevant relations between intelligence and personality, and several empirical findings support this conclusion. However, failures to find such relations might also be attributed to “the lack of appropriate methods for assessing personality-intelligence relations” [11]. In this regard, Stankov [13] suggests that we must increase the scope of the personality construct, moving beyond the lexical approach that characterizes the Big Five model. Kyllonen and Kell [12] show that intelligence is involved in personality assessment (and the other way around): “conclusions regarding the ability-personality relationship have as much to do with measurement methods as with construct similarities and differences”. Thus, we can conclude that it is worthwhile to reconsider the actual breadth of constructs considered, as well as the way they are assessed.

There are many interesting details in the articles of this Special Issue, and they deserve close attention. The interchange of comments derived from the complex and thought-provoking report by Demetriou et al. [15] is a perfect example of what we mean here. While the target article concludes that ‘cognizance’ (a set of processes including “self-monitoring of mental and behavioral processes, representation and awareness of them, reflection on and intentional regulation of them, and meta-representation, which generates new mental and behavioral constructs out of the modification and integration of available constructs”) might coordinate general intelligence and general personality, the comments by Ackerman [16] and Bäckström [17] raise several doubts and reservations, both at the conceptual and at the methodological level. These discussions are highly relevant for moving the field forward. The same conclusion can probably be drawn with regard to the role of the so-called general factor of personality. While such a general factor is seemingly ubiquitous in structural models of intelligence, its existence in personality questionnaire data is, to put it mildly, highly controversial. As the work by Kretzschmar et al. [9] in this Special Issue shows, the level of abstraction influences the relation estimates. Thus, we need conceptual clarity when moving further ahead.

Nevertheless, we are afraid that, after reviewing this set of articles and thinking about the implications, researchers will come back to their usual practice of focusing on a limited set of psychological constructs analyzed in isolation. Attempts to integrate intelligence and personality constructs have a very long tradition in psychological research, but we still lack the type of consensus we already have for the most likely structure of intelligence or the key components of human personality for a joint taxonomy (see DeYoung’s work [18]).

Joseph Royce and Arnold Powell published an ambitious and comprehensive theory in 1983 [19] aimed at integrating cognition and personality variables (the multifactor system theory of personality and individual differences). Philip Ackerman proposed the Process–Personality–Interests–Knowledge (PPIK) theory in 1996 [20]. Ziegler et al. proposed the OFCI model in 2012 [21]. Frank Schmidt’s integrative theory was published in 2014 [22]. Interestingly, the number of presumably relevant intelligence and personality factors has grown increasingly smaller over the years: from approximately 185 in Royce and Powell’s theory [19] to nine in Schmidt’s model [22]. There might be strong theoretical and empirical reasons underlying this trend, but the most troubling conclusion is the lack of impact of these proposals on everyday psychological research.

The relevance of relations between intelligence and personality for understanding human behavioral differences fits nicely with the summary by Sackett et al. [2] regarding personality constructs: “personality’s ride through the decades has not been as smooth as the one taken by cognitive ability, knowledge, and skills. Personality’s research history looks more a rollercoaster”.

Now that we are leaving the first quarter of the 21st century, we have a new chance to fix the problem and leave the rollercoaster for good. The editors of this Special Issue had this goal in mind when they first pondered this topic. Let us see if we can overcome the challenge this time.
